# Extra N-Terminal Residues Have a Profound Effect on the Aggregation Properties of the Potential Yeast Prion Protein Mca1

**DOI:** 10.1371/journal.pone.0009929

**Published:** 2010-03-29

**Authors:** Marc Erhardt, Renee D. Wegrzyn, Elke Deuerling

**Affiliations:** 1 Molekulare Mikrobiologie, Fachbereich Biologie, Universität Konstanz, Konstanz, Germany; 2 Zentrum für Molekulare Biologie der Universität Heidelberg (ZMBH), DKFZ-ZMBH Alliance, Universität Heidelberg, Heidelberg, Germany; University of Kent, United Kingdom

## Abstract

The metacaspase Mca1 from *Saccharomyces cerevisiae* displays a Q/N-rich region at its N-terminus reminiscent of yeast prion proteins. In this study, we show that the ability of Mca1 to form insoluble aggregates is modulated by a peptide stretch preceding its putative prion-forming domain. Based on its genomic locus, three potential translational start sites of Mca1 can give rise to two slightly different long Mca1 proteins or a short version, Mca1_451/453_ and Mca1_432,_ respectively, although under normal physiological conditions Mca1_432_ is the predominant form expressed. All Mca1 variants exhibit the Q/N-rich regions, while only the long variants Mca1_451/453_ share an extra stretch of 19 amino acids at their N-terminal end. Strikingly, only long versions of Mca1 but not Mca1_432_ revealed pronounced aggregation *in vivo* and displayed prion-like properties when fused to the C-terminal domain of Sup35 suggesting that the N-terminal peptide element promotes the conformational switch of Mca1 protein into an insoluble state. Transfer of the 19 N-terminal amino acid stretch of Mca1_451_ to the N-terminus of firefly luciferase resulted in increased aggregation of luciferase, suggesting a protein destabilizing function of the peptide element. We conclude that the aggregation propensity of the potential yeast prion protein Mca1 *in vivo* is strongly accelerated by a short peptide segment preceding its Q/N-rich region and we speculate that such a conformational switch might occur *in vivo* via the usage of alternative translational start sites.

## Introduction

Several proteins that can undergo structural conversion from a soluble state into an insoluble heritable prion conformation have been characterized in the yeast *Saccharomyces cerevisiae* including Sup35 forming the prion [*PSI^+^*] or Ure2p and its prion state [*URE3*] [1]–[5]. A common feature of these yeast prion proteins is the prion-forming domain (PFD), a glutamine and/or asparagine-rich (Q/N-rich) region with repeats of oligopeptides, such as the imperfect PQGGYQQYN repeats found in Sup35.

Sup35 is a translation termination factor and conversion to the [*PSI^+^*] prion state inactivates Sup35 molecules thereby increasing the levels of nonsense suppression [1], [6], [7]. The Sup35 protein has three domains, an N-terminal PFD which is essential for the conversion into the [*PSI^+^*] prion form, a middle domain and a C-terminal domain, which is essential for its function in translation termination [5], [8], [9].

The *in vivo* assay for monitoring [*PSI^+^*] usually involves read-through of nonsense alleles in auxotrophic markers, e.g. *ade1–14* (UAG) or *ade2-1* (UAA). Cells lacking the [*PSI^+^*] prion fail to synthesize adenine in the presence of a nonsense allele like *ade1–14*. Accordingly, [*psi^−^*] cells cannot grow on minimal media lacking adenine and in addition accumulate a red pigment on rich media under adenine-limiting conditions. [*PSI^+^*] cells, however, grow on minimal media lacking adenine (-ADE) and do not accumulate the red pigment on rich media [1], [10].

**Figure 1:**
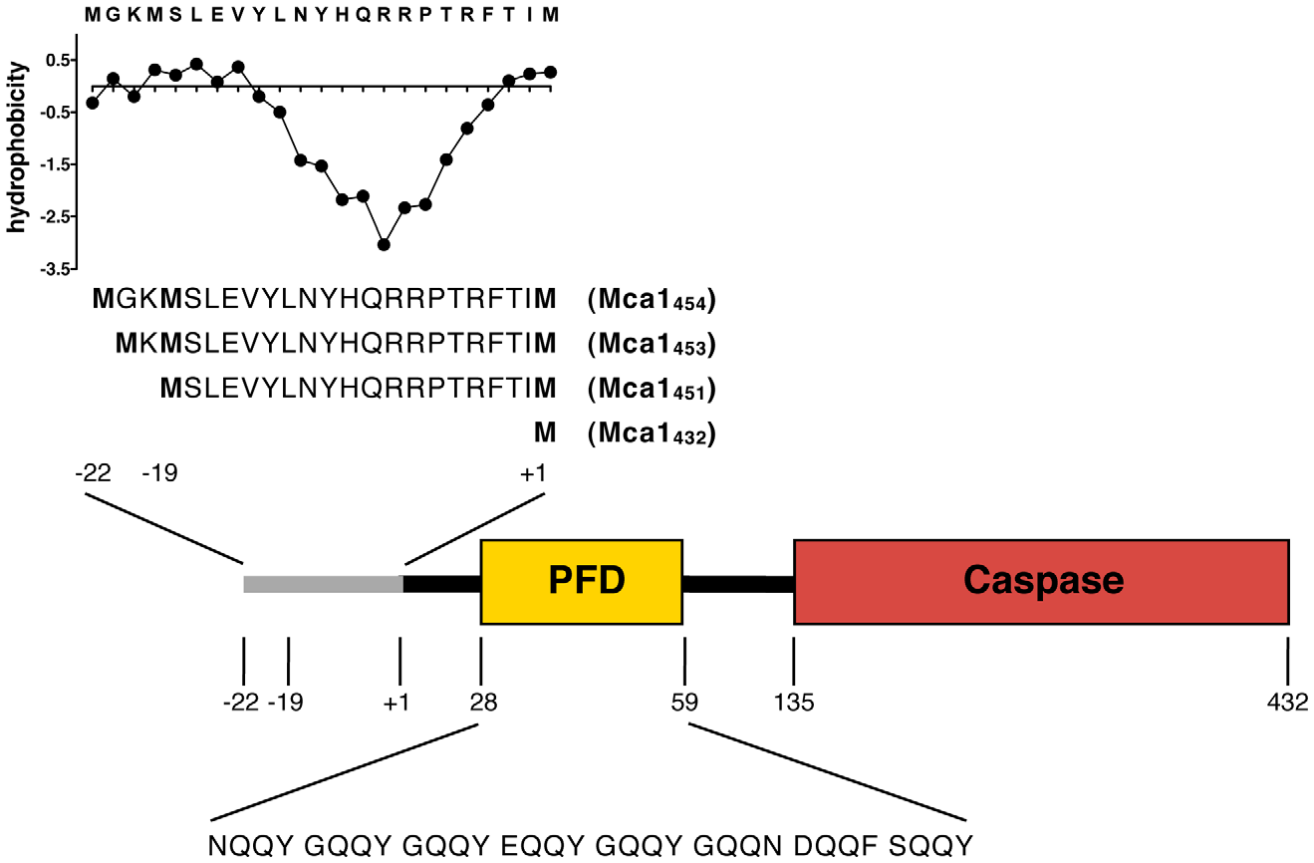
Domain architecture of metacaspase Mca1. The annotated metacaspase Mca1 consists of 432 amino acids (Mca1_432_), whereas previously two upstream translational start sites (57 and 63 base pairs upstream) have been annotated that give rise to Mca1 proteins of 451 amino acids (Mca1_451_, N-terminal extension of MSLEVYLNYHQRRPTRFTI) and 453 amino acids length (Mca1_453_, N-terminal extension of MKMSLEVYLNYHQRRPTRFTI). In this study, we additionally used a 454 amino acids long Mca1 protein (Mca1_454_, N-terminal extension of MGKMSLEVYLNYHQRRPTRFTI) that introduced an additional glycine residue after the methionine because of cloning considerations as outlined in the text and Materials and Methods. The additional N-terminal amino acids of the long Mca1 variants display a hydrophilic-hydrophobic charge distribution as shown in the upper left part of the figure. The hydrophobicity of the MGKMSLEVYLNYHQRRPTRFTIpeptide stretch was calculated using the ProtScale tool of the ExPASy Proteomics Server (www.expasy.org) that uses an amino acid scale described by Kyte and Doolittle [28]. All Mca1 variants also contain a Q/N-rich region reminiscent of the prion-forming domain (PFD) of Sup35 and other yeast prion proteins. Additionally, the putative PFD of Mca1 consists of several imperfect repeats of QQYGas visualized in the figure.

Recent database analyses and genetic screens were performed in order to detect new yeast proteins with key prion features including Q/N-rich regions, similar to prion forming domains of other yeast prions [11]–[13]. Nemecek et al. [13] detected Mca1 as a potential new prion protein by a genetic screen. Mca1 was described earlier as a metacaspase that regulates apoptosis in *Saccharomyces cerevisiae* [14]. Mca1 harbors a Q/N-rich region in its N-terminal domain that is characteristic for yeast prion proteins and aggregation-prone proteins. In addition, this putative prion-forming domain of Mca1 contains several imperfect repeats of QQYG that are reminiscent of the imperfect PQGGYQQYNrepeats found in the yeast prion protein Sup35 (Figure 1). Nemecek et al. [13] fused random yeast DNA fragments to the middle and C-terminal domain of Sup35 (Sup35MC) and selected for clones with increased read-through of the *ade2-1* nonsense allele caused by elevated aggregation of the Sup35MC fusion protein. Three different clones contained parts of the Q/N-rich N-terminal domain of Mca1 and the Ade^+^ phenotype of the Mca1-Sup35MC fusion protein dominantly segregated as a non-chromosomal genetic element, typical for prion proteins. Furthermore, the authors investigated several characteristic properties of yeast prions including curability and metastability. They found that the Ade^+^ phenotype could be cured by overexpression of Hsp104 and showed the reversible curability by spontaneous appearance of the prion protein in a previously cured strain. *De novo* formation of the Mca1 prion protein occurred by overexpression of the N-terminal domain of Mca1 suggesting that the isolated Ade^+^ clone, which contained a portion of Mca1, fulfills the criteria of a yeast prion [13]. In another recent study, Alberti et al. performed a bioinformatic proteome-wide survey for proteins with prion-like properties in *S. cerevisiae* [11]. The authors experimentally investigated 100 prion protein candidates and found 19 new prions. Also in this study, Mca1 had been tested for its aggregation properties, albeit the authors did not find compelling evidence for [*MCA^+^*] being a prion. Mca1 did neither form SDS-resistant aggregates in semi-denaturing detergent-agarose gel electrophoresis, which would indicate prion-like structures, nor did a Mca1-Sup35C fusion protein display an Ade^+^ phenotype.

Interestingly, based on its genetic structure three potential translational starts of the *MCA1* gene from yeast (*YOR197W*) exist which could give rise to different isoforms. Two potential translational start sites of *MCA1* are in immediate vicinity giving rise to Mca1 proteins that differ only slightly by two amino acids in length (453 and 451 amino acids, respectively), while the third one is more distant giving rise to a significantly shorter Mca1 protein of 432 amino acids. Which of these potential translational start sites are used *in vivo* is still unclear due to the lack of supportive experimental data. In addition, the annotated translational start of *MCA1* was changed over the past years. Based on automated genome sequence comparison of closely related *Saccharomyces* species, the translational start site of *MCA1* was recently moved downstream to the third AUG, giving rise to the short version of the Mca1 protein (432 amino acids) [15], [16].

Here we show that dependent on which isoform of Mca1 is expressed, the aggregation propensity of Mca1 is strongly affected. While the short version Mca1_432_ is completely soluble, the long versions of Mca1 aggregate *in vivo*. The conversion of Mca1 from a soluble protein into an insoluble conformation with prion-like properties is triggered by the additional peptide stretch present at the N-terminus of the long Mca1 versions. However, under regular growth conditions only the short soluble isoform is detectable *in vivo*.

## Results

### Mca1_432_ is predominantly expressed *in vivo*

We were intrigued by the possibility of Mca1 from *S. cerevisiae* being a potential new yeast prion protein and set out to examine the aggregation and prion properties of this protein more closely. The domain architecture and the putative prion-forming domain (PFD) of Mca1 are displayed in Figure 1. First, we determined which potential initiation codon of the *MCA1* allele is used for translation *in vivo*. The use of the recently annotated start would result in a Mca1 protein of 432 amino acids (designated hereafter Mca1_432_), while the use of the AUG start codons 57 or 63 nucleotides upstream of the currently annotated AUG would result in a Mca1 protein of 451 or 453 amino acid residues, respectively (Figure 1). Interestingly, homologous Mca1 proteins from other fungi display similar features in respect to their translational start site. For example, *Kluyveromyces lactis* Mca1 contains a Q/N-rich N-terminal region and a potential second translational start site, giving rise to a 20 amino acids N-terminally extended isoform (data not shown).

**Figure 2:**
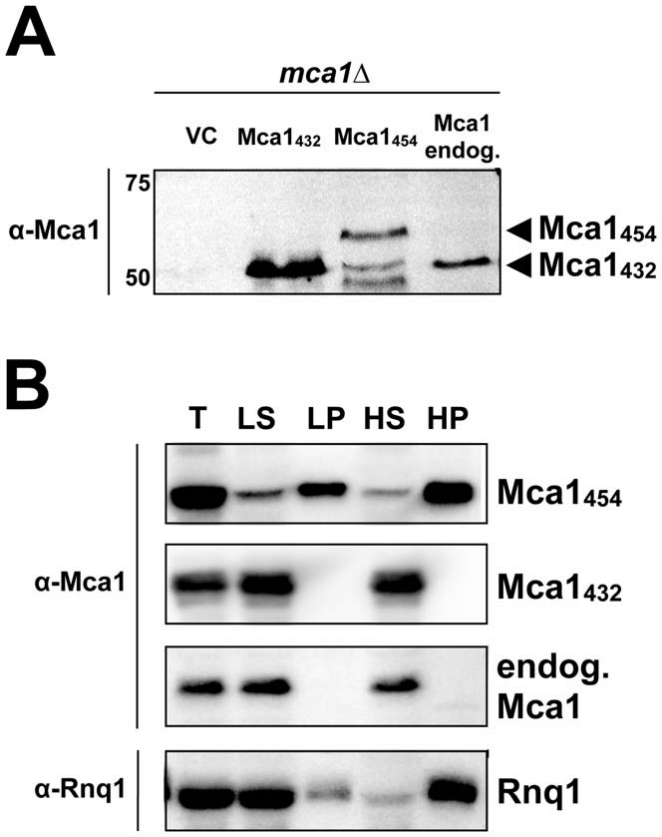
Native start site and aggregation analysis of Mca1. (A) Mca1_432_ and Mca1_454_ (N-terminal extension MGKMSLEVYLNYHQRRPTRFTI) were expressed from a copper-inducible promoter in the presence of 150 µM CuSO4 and endogenous Mca1 was expressed from its native promoter in a strain lacking the chromosomal *MCA1* gene (Y103). Mca1 protein was detected using polyclonal anti-Mca1 antibodies and immunoblotting. Plasmids used in this assay: V294 (vector control  =  VC), V413 (Mca1_432_), V414 (Mca1_454_) and V415 (endogenous Mca1). (B) *MCA1*_432_ and *MCA1*_454_ (N-terminal extension MGKMSLEVYLNYHQRRPTRFTI) were expressed from a copper-inducible promoter and endogenous Mca1 was expressed from its native promoter in a *mca1Δ* strain (Y103). Soluble and aggregated proteins were separated by low-spin (18,000×g) and high-spin (100,000×g) centrifugation. Mca1 protein was detected using polyclonal anti-Mca1 antibodies (raised against Mca1_454_) and immunoblotting. Rnq1 was detected using polyclonal anti-Rnq1 antibodies. (T) total lysate; (LS) low-spin supernatant fraction; (LP) low-spin pellet fraction; (HS) high-spin supernatant fraction; (HP) high-spin pellet fraction. Plasmids used in this assay: V294 (vector control  =  VC), V413 (Mca1_432_), V414 (Mca1_454_) and V415 (endogenous Mca1). Please note that the two smaller fragments of Mca1_454_ seen in A (see text for details) were also present in B, albeit not shown in this section.

The additional amino acids extension at the N-terminus of longer Mca1 versions should result in a molecular weight difference of 2.4 or 2.7 kDa compared to Mca1_432_. However, PFD containing proteins migrate aberrantly in SDS-PAGE gels [17]–[19]. Accordingly, we could not accurately determine the molecular weight of Mca1 expressed *in vivo* by immunoblotting of wild type lysate with polyclonal antibodies raised against Mca1. Also, our attempts to purify C-terminally TAP-tagged Mca1 expressed under authentic chromosomal conditions from *S. cerevisiae* failed due to the very low abundance of Mca1 and massive contamination by unspecifically co-purified proteins which did not allow the determination of its molecular mass by mass spectrometry. Therefore, we chose an alternative strategy to elucidate the translational start site used *in vivo*. We cloned two *MCA1* genes with different translational start sites that served as standards to distinguish the long Mca1 versions from the short Mca1 protein. The short *MCA1* gene contained only the third AUG (bp +1 to bp +1299 respective to the currently annotated *MCA1* coding sequence) giving rise to Mca1_432_. The long version of *MCA1* started at the first AUG (bp −63 to bp +1299 respective to the annotated *MCA1* coding sequence). Due to cloning considerations the long *MCA1* gene encoded an additional glycine residue after the initial methionine thereby resulting in a Mca1 protein of 454 amino acids (designated hereafter Mca1_454,_ Figure 1). Both *MCA1* genes were cloned under control of a copper-inducible promoter into pRS313. To determine the authentic translational start of *MCA1 in vivo*, we additionally cloned the *MCA1* containing DNA segment including its endogenous promoter and terminator regions (*MCA1*_endog_  = bp −582 to bp +1799 respective to the annotated *MCA1* coding sequence) into pRS313 yeast vector lacking the copper-inducible promoter. We transformed the plasmids encoding *MCA1*_454_ (V414) and *MCA1*_432_ (V413), as well as *MCA1* under endogenous control (*MCA1*_endog_  = V415) into *mca1Δ* yeast cells lacking the chromosomal *MCA1* gene. After copper induction of the plasmid encoded *MCA1*_454_ and *MCA1*_432_ genes, total cell lysates were prepared and all Mca1 variants were visualized by immunodetection using polyclonal Mca1 antibodies. As shown in Figure 2A, both Mca1 isoforms could be distinguished by their migration behavior in SDS-PAGE albeit they only displayed a small mass difference (Figure 2A, lanes 2 and 3). While cells expressing Mca1_432_ showed only one signal with an approximate size of 50 kDa, cells expressing Mca1_454_ driven by the copper promoter showed a signal at a size of about 55 kDa reflecting full-length Mca1_454_ and two additional smaller products. We assume that the smaller products are either due to the usage of alternative downstream AUG translational start sites or perhaps resulted from proteolytic degradation. Importantly, comparison with the signal obtained from cells expressing *MCA1* under endogenous control clearly showed that the short version Mca1_432_ is identical to wild type Mca1 indicating that the third translational start site of *MCA1* is predominantly used *in vivo*.

### Aggregation of Mca1 is dependent on the N-terminal amino acid residues preceding the putative prion-forming domain

Importantly, the putative prion-forming domain is present in both cloned Mca1 variants, Mca1_454_ and Mca1_432_. Thus, we analyzed the aggregation properties of the different Mca1 isoforms by preparing lysates from *mca1Δ* cells expressing copper-inducible *MCA1*_454_ or *MCA1*_432_, as well as *MCA1* under endogenous control. The lysates were applied to sequential centrifugation analysis to differentiate between large aggregates that sediment by low-speed centrifugation, and smaller ones or aggregates with specific sedimentation properties such as Rnq1 aggregates that could be monitored by high-speed centrifugation. As it is evident in Figure 2B, endogenous Mca1 and the short version Mca1_432_ controlled by the copper-inducible promoter were exclusively found in the supernatant but not in the pellet fraction implying that these Mca1 variants do not aggregate under the tested conditions. In contrast, the Mca1_454_ variant revealed insoluble material by both, low-speed and high-speed centrifugation with 18,000 g and 100,000 g, respectively. Based on the fact that the same promoter drives both Mca1 variants, Mca1_432_ and Mca1_454_, and that we detected similar total protein levels, the pronounced difference in the solubility can be attributed to the additional N-terminal amino acid stretch present in Mca1_454_.

**Figure 3:**
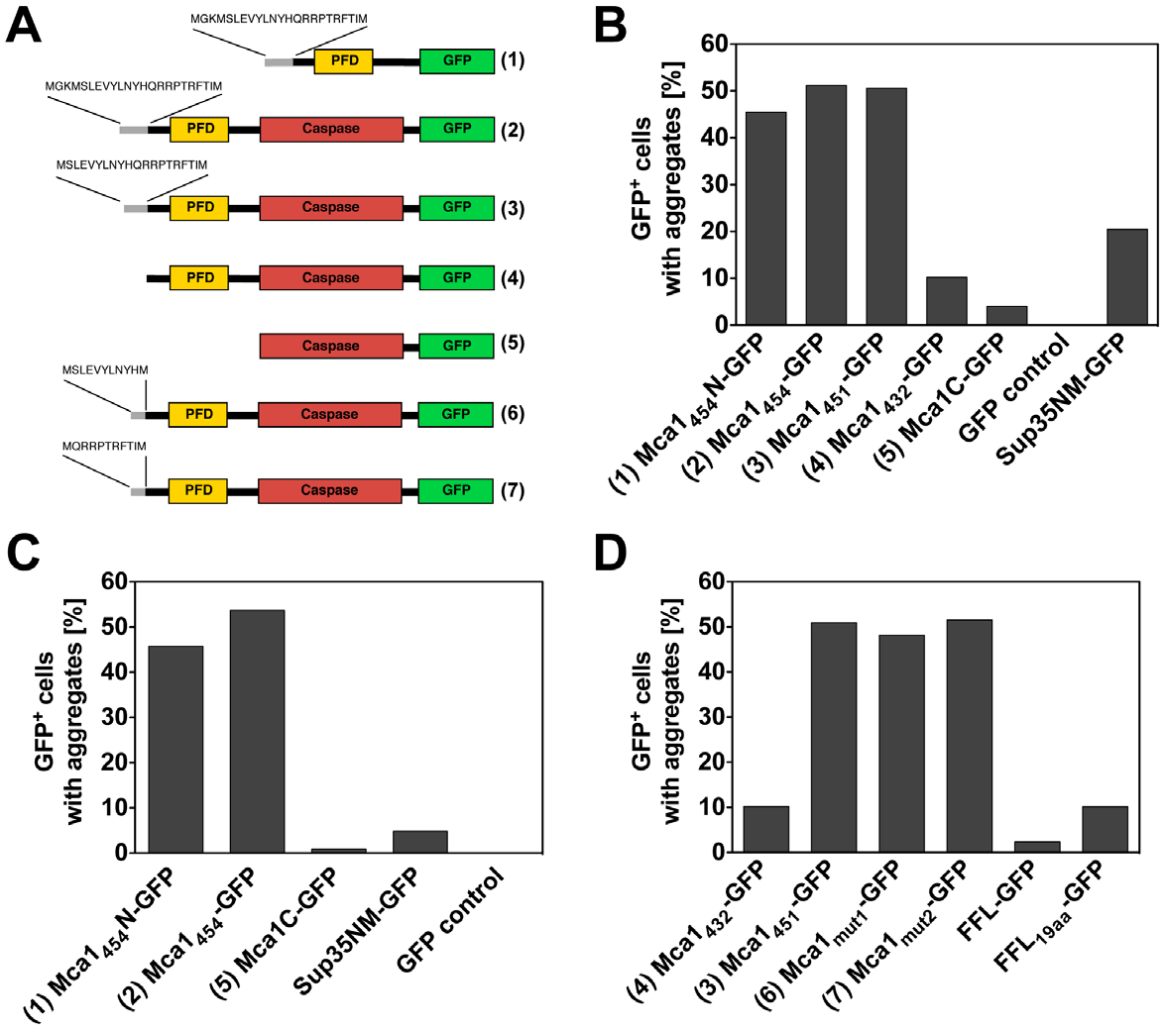
Quantification of Mca1-GFP aggregates. (A) Domain architecture of the various Mca1-GFP fusion constructs used for fluorescence microscopy analysis. (1) Mca1_454_N-GFP, contains the N-terminal extension MGKMSLEVYLNYHQRRPTRFTIand the prion-forming domain of Mca1_454_ (amino acids 1-148) fused to sGFP; (2) Mca1_454_-GFP, Mca1 with an N-terminal extension of MGKMSLEVYLNYHQRRPTRFTIfused to sGFP; (3) Mca1_451_-GFP, Mca1 with an N-terminal extension of MSLEVYLNYHQRRPTRFTIfused to sGFP; (4) Mca1_432_-GFP; (5) Mca1C-GFP, caspase domain of Mca1 fused to sGFP; (6) Mca1_mut1_-GFP, Mca1 with an N-terminal extension of MSLEVYLNYHfused to sGFP, and (7) Mca1_mut2_-GFP, Mca1 with an N-terminal extension of MQRRPTRFTI fused to sGFP. (B) Mca1_454_N-GFP, Mca1_454_-GFP, Mca1_451_-GFP, Mca1_432_-GFP, Mca1C-GFP, Sup35NM-GFP and GFP control were expressed in a strain lacking the chromosomal *MCA1* gene (Y103) for 24 hours by induction with 150 µM CuSO4. GFP-expressing cells were analyzed using fluorescence microscopy. Plasmids used: V454 (Mca1_432_-GFP), V455 (Mca1_451_-GFP), V84 (Mca1_454_N-GFP), V85 (Mca1_454_-GFP), V106 (Mca1C-GFP), V66 (Sup35NM-GFP) and V26 (GFP control). (C) Mca1_454_N-GFP, Mca1_454_-GFP, Mca1C-GFP, Sup35NM-GFP and GFP control were expressed in a *MCA1^+^* (WT  =  Y67) strain by induction with 150 µM CuSO4 for 24 hours. GFP-expressing cells were analyzed using fluorescence microscopy. Plasmids used in this assay: V84 (Mca1_454_N-GFP), V85 (Mca1_454_-GFP), V106 (Mca1C-GFP), V66 (Sup35NM-GFP) and V26 (GFP control). (D) Mca1_432_-GFP, Mca1_451_-GFP, Mca1_mut1_-GFP, Mca1_mut2_-GFP, firefly luciferase - GFP (FFL-GFP) and FFL-GFP with the 19 amino acids N-terminal extension of MSLEVYLNYHQRRPTRFTI(FFL_19aa_-GFP) were expressed in a *mca1Δ* strain (Y103) by induction with 150 µM CuSO4 for 24 hours. GFP-expressing cells were analyzed using fluorescence microscopy. Plasmids used in this assay: V454 (Mca1_432_-GFP), V455 (Mca1_451_-GFP), V456 (Mca1_mut1_-GFP), V457 (Mca1_mut2_-GFP), V481 (FFL-GFP) and V458 (FFL_19aa_-GFP).

**Figure 4:**
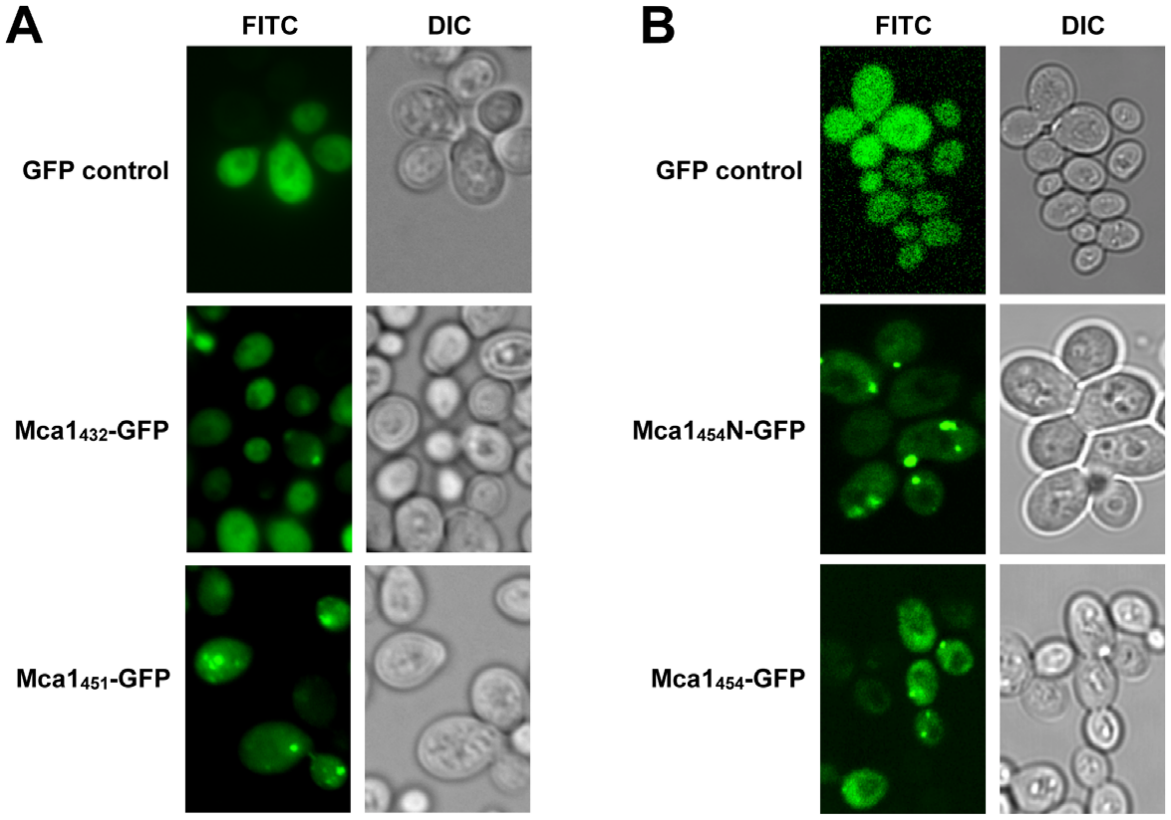
Fluorescence microscopy of Mca1-GFP aggregates. (A) Strain Y103 (*mca1Δ*) expressing copper-inducible GFP control, Mca1_432_-GFP and Mca1_451_-GFP was analyzed by fluorescence microscopy after 24 hours of induction with 150 µM CuSO4. Punctate Mca1-GFP aggregates are predominantly found in cells expressing Mca1_451_-GFP, but not in cells expressing Mca1_432_-GFP. DIC  =  differential interference contrast. (B) Strain Y75 (OT55; wildtype *MCA1*, weak [*PSI^+^*][*PIN^+^*]) expressing copper-inducible GFP control, Mca1_454_N-GFP and Mca1_454_-GFP was analyzed by fluorescence microscopy after 24 hours of induction with 150 µM CuSO4. Punctate GFP aggregates are predominantly found in cells expressing Mca1_454_-GFP and Mca1_454_N-GFP. DIC  =  differential interference contrast.

To further analyze the aggregation properties of Mca1 *in vivo*, we fused the different Mca1 constructs to green-fluorescent protein (GFP) under the control of a copper-inducible promoter (Figure 3A) and expressed the fusion proteins in cells lacking the chromosomal *MCA1* gene. As discussed above, three translational start sites can be theoretically engaged based on the genomic locus of *MCA1*. Thus, we also cloned the third Mca1_451_ variant as GFP-fusion protein (*MCA1*_451_  = bp −57 to bp +1299 respective to the annotated *MCA1* coding sequence, Figure 1) to test for the solubility of the second extended Mca1 version (Figure 3 and Figure 4).

The expression of both Mca1_454_-GFP and Mca1_451_-GFP fusion proteins led to aggregate formation after 24 hours of induction in cells deleted for the chromosomal *MCA1* gene as well as in wild type cells. In contrast, for the short version of Mca1 (Mca1_432_-GFP) and for the truncated Mca1 variant lacking the putative prion-forming domain (Mca1C-GFP) significantly less GFP foci were found (Figure 3 and Figure 4). A quantitative analysis of the frequency of aggregate formation in cells deleted for chromosomal *MCA1* revealed Mca1_454_-GFP and Mca1_451_-GFP aggregates in about 50% of the cells, whereas we found Mca1_432_-GFP aggregates in only about 10% of the analyzed cells (Figure 3B). Importantly, both long Mca1 variants, Mca1_454_-GFP and Mca1_451_-GFP, share the 19 amino acids N-terminal extension segment (MSLEVYLNYHQRRPTRFTI, Figure 1) and show very similar aggregation properties *in vivo*. Thus, the major difference in solubility observed for the two long Mca1 variants compared to the short Mca1_432_-GFP protein can be attributed to this N-terminal stretch of additional 19 amino acids which is absent in Mca1_432_-GFP. As control, we investigated the fusion protein Sup35NM-GFP which was expressed by similar means and at comparable levels (data not shown and Figure 3B). Expression of the Sup35NM-GFP fusion protein caused aggregation in about 20% of the cells which is consistent with previously published data [20] supporting the significance of Mca1_451_-GFP and Mca1_454_-GFP aggregation under the conditions we tested. We additionally analyzed the effects of Mca1_454_–GFP aggregation in cells containing a wild type copy of *MCA1* and found no difference in the frequency of GFP aggregate formation (Figure 3C). Importantly, a fusion comprising only the N-terminal extension of Mca1_454_ together with the putative prion-forming domain of Mca1 (Mca1_454_N-GFP containing amino acids 1 to 148 of Mca1_454_) to GFP displayed aggregation levels comparable to full length Mca1_454_ and Mca1_451_. In contrast, truncated Mca1 containing only the caspase domain (Mca1C, amino acids 150 to 451) fused to GFP did not aggregate (Figures 3B+3C) suggesting that the PFD domain is essential but not sufficient for the pronounced aggregation of Mca1_454_ and Mca1_451_.

In summary, we conclude that the short GFP-fusion version of Mca1_432_ has only a latent aggregation tendency. However, the addition of 19 amino acids to the N-terminus of Mca1_432_ converts the Mca1 protein into a strong aggregation-prone variant *in vivo*.

Next, we analyzed the N-terminal 19 amino acids of Mca1 for their biochemical properties and found an unusual hydrophobic-hydrophilic charge distribution (Figure 1). We wondered whether the strong hydrophobic character within the first 10 amino acids is responsible for the strongly enhanced aggregation of Mca1_451_. Therefore, we divided the 19 amino acids into two parts and fused either the hydrophobic (Mca1_mut1_) or the hydrophilic part (Mca1_mut2_) of the 19 amino acids stretch to Mca1_432_-GFP and analyzed the different truncation mutants for their ability to form GFP aggregates. As displayed in Figure 3D, the frequency of aggregate formation was not altered by the different truncations, indicating that the presence of either hydrophilic or hydrophobic stretch at the N-terminus of the prion-forming domain of Mca1 is sufficient to destabilize the protein conformation.

Next, we investigated whether the destabilizing effect of these 19 amino acids is specific for Mca1 or perhaps portable to other proteins as well. To this end, we constructed a fusion protein containing the N-terminal 19 amino acids stretch of Mca1_451_ fused N-terminally to firefly luciferase together with a GFP moiety at the C-terminus (FFL19_aa_-GFP). Luciferase has no Q/N-rich region and displayed only minor protein aggregation on its own. However, the frequency of GFP aggregates was 3-fold increased for FFL19_aa_-GFP although the aggregation was clearly less pronounced compared to Mca1_451_ (Figure 3D). We conclude that the increased ability to form aggregates of Mca1_451_ and FFL19_aa_ is due to the presence of the additional N-terminal 19 amino acids stretch that presumably destabilizes protein conformations in general but shows a more dramatic effect when combined with the Q/N-rich region of Mca1.

### The extended version of Mca1 fused to Sup35C displays a nonsense-suppressor phenotype

Intrigued by the ability of the long Mca1 isoforms to form aggregates dependent on their extra N-terminal amino acids and the Q/N-rich prion-forming domain, we examined the aggregation properties and a potential prion-like behavior of Mca1_454_ more closely. In order to mimic the suppressor phenotype of [*PSI^+^*], the prion isoform of the translation termination factor Sup35, we constructed different Mca1_454_ fusion proteins to the C-terminal translation termination domain of Sup35 (Sup35C). It is important to note that all Mca1_454_-Sup35C fusion proteins did complement a *sup35Δ* deletion strain indicating that the Mca1_454_-Sup35C fusions are functional *in vivo* (not shown).

**Figure 5:**
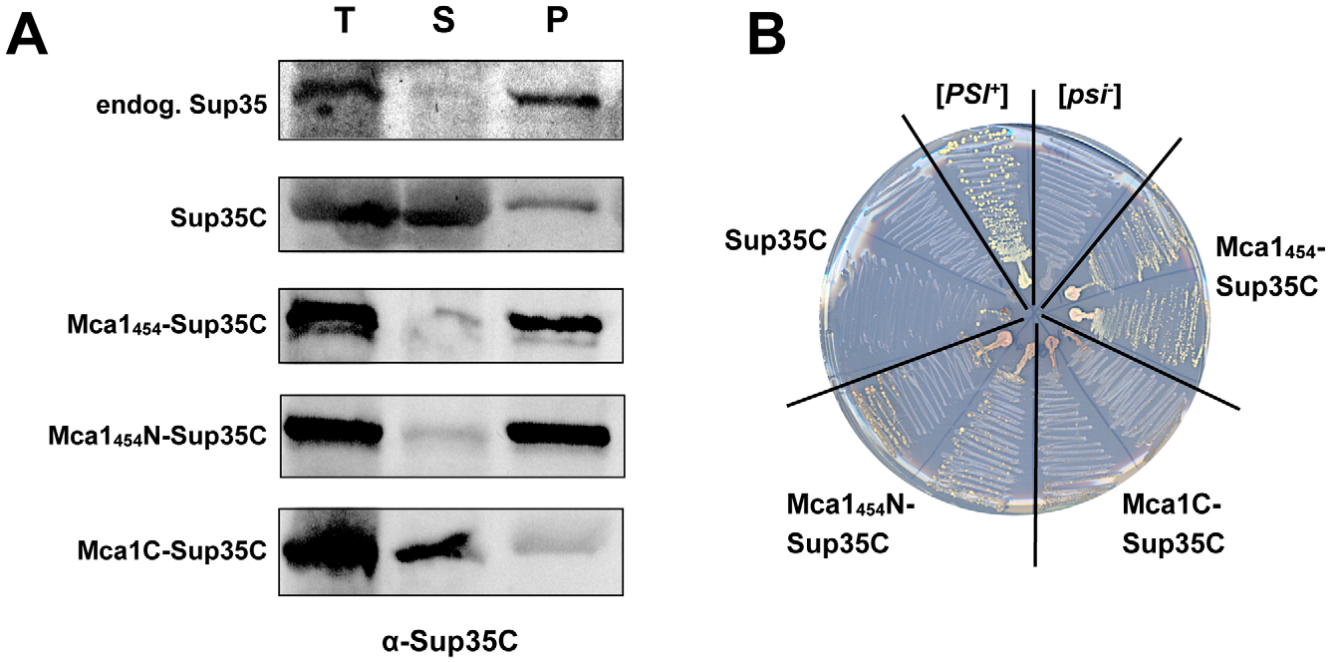
Aggregation analysis and Ade^+^ phenotype of Mca1_454_-Sup35C. (A) Full-length Mca1_454_ (N-terminal extension of MGKMSLEVYLNYHQRRPTRFTI), the N-terminal extension and Q/N-rich region of Mca1_454_ (Mca1_454_N) and Mca1C (caspase domain of Mca1) were fused to Sup35C and expressed in a *sup35Δ* strain. Soluble and aggregated proteins were separated by centrifugation analysis and detected using polyclonal anti-Sup35C antibodies and immunoblotting. Mca1_454_-Sup35C fusions revealed aggregation dependent on the Q/N-rich region of Mca1_454_. Strains used in this assay (from top to bottom): Y133 (endogenous Sup35p; [*PSI^+^*]), Y312 (p2HG-*SUP35C*), Y320 (p2HG-*MCA1_454_-SUP35C*), Y316 (p2HG-*MCA1_454_N-SUP35C*), Y322 (p2HG-*MCA1C-SUP35C*). (B) Full-length Mca1_454_ (N-terminal extension of MGKMSLEVYLNYHQRRPTRFTI), the N-terminal extension and Q/N-rich region of Mca1_454_ (Mca1_454_N) and Mca1C (caspase domain of Mca1) were fused to Sup35C and constitutively expressed in a *sup35Δ* strain harboring the chromosomal *ade1–14* mutation. Growth of two independent clones was analyzed on media lacking adenine after 12 days of incubation. Strains used in this assay: Y133 (endogenous Sup35p; [*PSI^+^*]), Y81 ([*psi^−^*][*pin^−^*]), Y320 (p2HG-*MCA1_454_-SUP35C*), Y316 (p2HG-*MCA1_454_N-SUP35C*), Y322 (p2HG-*MCA1C-SUP35C*), Y312 (p2HG-*SUP35C*).

First, we studied the ability of different Mca1_454_-Sup35C fusion proteins to form protein aggregates by centrifugation analysis. As shown in Figure 5A, the aggregate formation of Mca1_454_-Sup35C was dependent on the Q/N-rich region of Mca1_454_. Neither Sup35C alone, nor Mca1C-Sup35C that lacks the entire N-terminal domain including the PFD of Mca1 displayed pronounced aggregation properties. However, we detected strong aggregation of both full-length Mca1_454_-Sup35C and a variant containing the N-terminal extension of Mca1_454_ together with the PFD-domain (Mca1_454_N-Sup35C) in the respective pellet fractions. Importantly, the aggregation properties of both Mca1_454_-Sup35 and Mca1_454_N-Sup35C closely mimic the aggregation properties of endogenous Sup35 in a [*PSI^+^*] strain (Figure 5). These results indicate that Mca1_454_ is able to mimic the aggregation-prone nature of Sup35 prion proteins *in vivo*.

To investigate this possibility further, we examined the ability of the different Mca1_454_- Sup35C fusion proteins to induce an Ade^+^ phenotype of a strain containing the *ade1–14* nonsense mutation. We generated strains lacking chromosomally encoded *SUP35* but expressing the Mca1_454_-Sup35C, Mca1_454_N-Sup35C, or Mca1C-Sup35 fusion protein from a constitutive GPD-promoter, and tested two independently isolated clones of each strain. While cells expressing the Mca1C-Sup35 fusion protein or only Sup35 showed no growth, cells harboring the Mca1_454_-Sup35C or Mca1_454_N-Sup35C fusion protein showed growth on adenine minimal media (Figure 5B) and also white color on rich media (data not shown) confirming the pronounced aggregation properties of Mca1_454_.

In summary, our results suggest that the prion-like domain of Mca1, together with the extra N-terminal peptide stretch of Mca1_454_, has the capacity to convert Mca1 into an aggregation-prone state that can additionally induce an Ade^+^ nonsense suppressor phenotype similar to the Sup35 prion protein.

## Discussion

The metacaspase Mca1 of *Saccharomyces cerevisiae* contains a Q/N-rich region similar to the prion-forming domains of yeast prion proteins like Sup35. Mca1 can theoretically be expressed in three isoforms that differ by extra amino acids at the N-terminus. In this study, we showed that this N-terminal extension segment is critical for the pronounced aggregation properties of Mca1. We found that the longer versions of Mca1 (Mca1_451/454_), which share an N-terminal extension of 19 amino acids, form significantly more aggregates than the shorter version of Mca1 (Mca1_432_). We further analyzed the aggregation properties of Mca1_432_, Mca1_451_, Mca1_454_ and various truncation mutants of the N-terminal 19 amino acids stretch upstream of the endogenous start site and found that the ability of Mca1 to form aggregates is dependent on the addition of upstream N-terminal amino acids as well as on the presence of the Q/N- rich region of Mca1.

To our surprise, the length and the overall character of the N-terminal extension seems not to be of decisive importance for the aggregation of Mca1. The frequency of aggregate formation was similar for the two extended Mca1 versions (Mca1_454_ and Mca1_451_) and also for the truncation mutants of the amino-terminal hydrophobic-hydrophilic stretch, Mca1_mut1_ (containing the hydrophobic part) and Mca1_mut2_ (containing the hydrophilic part). This indicates that the addition of either a hydrophobic, hydrophilic, or hydrophobic-hydrophilic stretch to the N-terminus of the Q/N-rich region of Mca1 is sufficient for destabilizing the protein conformation. The addition of the hydrophobic-hydrophilic 19 amino acids stretch to the N-terminus of firefly luciferase also increased the frequency of aggregate formation. Firefly luciferase lacks an aggregation-prone Q/N-rich region, suggesting that the 19 amino acids N-terminal extension has a general destabilizing effect on protein conformations. Such destabilizing effects of N-terminal extensions had been observed in previous studies investigating polyQ-proteins, which are not related to prions but also capable to convert their structures into amyloid-like fibers. A FLAG-tag was found to unmask the latent polyQ length-dependent toxicity in polyQ-expanded exon I of Huntingtin protein (Htt) [21]. Moreover, a very recent analysis of the Frydman lab showed that polyQ aggregation kinetics is not solely a function of polyQ repeat length, but rather includes the critical contribution of the N-terminal 17 amino acid residues forming an amphipathic helix and promoting rapid Htt aggregation by direct modulation of Htt conformation [22]. Thus far, it is unclear why and how the N-terminal extension of Mca1 has such a dramatic impact on the aggregation properties of Mca1 and further analyses are required to resolve that finding on a mechanistic basis. However, we speculate that the addition of the hydrophobic-hydrophilic 19 amino acids stretch to the N-terminus of Mca1 or luciferase presumably destabilizes the protein conformation, thereby increasing the probability of aggregate formation, which in case of Mca1 allows for conversion of the protein into an aggregation-prone state potentially displaying prion-like properties.

Intrigued by the possibility that the sole addition of N-terminal amino acids might trigger the aggregation properties of Mca1, we furthermore characterized the potential of the aggregation-prone version of Mca1 (Mca1_454_) to induce an Ade^+^ phenotype if fused to the C-terminal domain of Sup35. We found that Mca1_454_-Sup35C fusion constructs can complement a *sup35Δ* deletion strain and indeed displayed an Ade^+^ phenotype dependent on the putative prion-forming domain of Mca1_454_. Only constructs containing the Q/N-rich domain of Mca1_454_ fused to Sup35C were able to suppress the adenine deficient phenotype of strains harboring the *ade1–14* mutation, as analyzed by growth on adenine minimal media as well as white color on rich media. Thus, the long Mca1 isoform, Mca1_454_, reveals an aggregation behavior reminiscent of yeast prion proteins like Sup35.

The question remains why Nemecek et al. [13] detected Mca1 as a yeast prion protein in their genetic screen, whereas Alberti et al. [11] did not detect Mca1 in their respective systematic screen for yeast prions. Based on the results presented in this study, we can speculate about this issue. Nemecek and coworkers [13] investigated a *MCA1* fragment starting 161 base pairs upstream of the *MCA1* coding region including all potential translation initiation sites fused to Sup35C on a plasmid called p20MCA. Thus, in addition to the shorter Mca1_432_ version, theoretically also the longer Mca1 version could be expressed by their construct, perhaps at a level that could not be detected under their assay conditions but sufficient to trigger aggregation of Mca1. In contrast, Alberti et al. [11] cloned *MCA1* for their study according to the currently annotated start site that is lacking the destabilizing N-terminal amino acids. This would resolve, together with our findings showing that the extra N-terminal amino acids modulate the aggregation properties of Mca1, the discrepancy in the conclusions regarding the prion properties of Mca1 that are present in the two studies [11], [13]. Alternatively, and not mutually exclusive, it is also possible that differences in the experimental systems used by Alberti et al. (who focused on full-length Mca1 fusions to reporter proteins) and Nemecek et al. (who fused the N-terminal proposed prion-forming domain of Mca1 to reporter proteins) could contribute to the different findings about the prion properties of Mca1.

Many intrinsic and extrinsic factors are known that can contribute to the conversion of soluble proteins into an aggregation-prone or prion-like state, including chaperones and various stress conditions. To our knowledge, this is the first report about a potential yeast prion protein that strongly varies in its aggregation and potential prion-like properties based on the translational start codon employed by nature. We provide evidence that the delicate balance of Mca1 conformation depends on its N-terminal start. Although there is no proof so far that such a translational switch of the start codon occurs *in vivo* for Mca1 or any other prion-like protein, we consider such a hypothesis as very attractive. It is tempting to speculate that perhaps yeast cells may also use the alternative translation initiation codons of *MCA1* under special environmental conditions. It is known that alternative usage of ATG codons can be triggered, for example, in a hormone-dependent manner in the testis or by stress conditions [23].

Thus far, we could not detect such a condition for Mca1 (data not shown). Interestingly, we found by screening yeast proteins harboring potential prion-forming domains for additional upstream start sites that Pgd1, a subunit of the RNA polymerase II mediator complex, also possesses an alternative, upstream start site. This finding indicates that other aggregation-prone proteins might also feature alternative start sites that may change their aggregation properties according to the isoform made in the cell. Additionally, Komar et al. [24] described an internal ribosome entry site (IRES) in the mRNA of *URE2*. Expression of Ure2 from that internal initiation site resulted in a truncated Ure2 protein that lacked the prion-forming domain. Importantly, the authors showed that this alternative, truncated Ure2 protein affected the [*URE3*] prion phenotype indicating that yeast cells can influence propagation of prion proteins by using different translation initiation sites depending on e.g. growth conditions or environmental signals.

## Materials and Methods

### Strains and plasmids used in this study

Strains and plasmids constructed and used in this study are listed in Table 1 and Table 2. Cloning strategies and primer sequences are listed in supplemental Table S1. The *sup35Δ* strain Y133 was generated by transforming strain Y119 [25] with PCR-generated copies of the *kanmx* cassette amplified from plasmid pFA6a-KanMX6 [26] with primers containing regions homologous to the *SUP35* locus: (CCATTGTACTGTAACAAAAAGCGGTTTCTTCATGACTTGCTCGGcggatccccgggttaattaa and GCATTTACTTATGTTTGCAAGAAATTTACTCGGCgaattcgagctcgtttaaac, regions homologous to *SUP35* locus indicated in capital letters).

**Table 1:** List of plasmids used and constructed in this study.

Plasmid number	Relevant characteristics	Vector backbone	Reference
V26 (pmCUPsGFP)	P_CUP1_-*sGFP*	pRS316	[7]
V29		pRS313	[29]
V66 (CNMsG)	P_CUP1_-*SUP35NM-sGFP*	pRS316	[7]
V84	P_CUP1_-*MCA1_454_N-sGFP* (amino acids 1-148 of Mca1_454_)	pRS316	this study
V85	P_CUP1_-*MCA1_454_-sGFP* (N-terminal extension of MGKMSLEVYLNYHQRRPTRFTI)	pRS316	this study
V106	P_CUP1_-*MCA1C-sGFP*	pRS316	this study
V119	P_GPD_	p2HG	[8]
V123	P_GPD_-*MCA1_454_-SUP35C* (N-terminal extension of MGKMSLEVYLNYHQRRPTRFTI)	p2HG	this study
V124	P_GPD_-*MCA1_454_N-SUP35C* (amino acids 1-148 of Mca1_454_)	p2HG	this study
V236	P_GPD_-*SUP35C*	p2HG	this study
V257	P_GPD_-*MCA1C-SUP35C*	p2HG	this study
V294	pmCUP313	pRS313	[30]
V334	P_CUP1_-*MCA1_432_N-sGFP*	pRS316	this study
V413	P_CUP1_-*MCA1_432_*	pRS313	this study
V414	P_CUP1_-*MCA1_454_* (N-terminal extension of MGKMSLEVYLNYHQRRPTRFTI)	pRS313	this study
V415	P_endo_-*MCA1*	pRS313	this study
V454	P_CUP1_-*MCA1_432_-sGFP*	pRS316	this study
V455	P_CUP1_-*MCA1_451_-sGFP* (N-terminal extension of MSLEVYLNYHQRRPTRFTI)	pRS316	this study
V456	P_CUP1_-*MCA1_mut1_-sGFP* (N-terminal extension of MSLEVYLNYH)	pRS316	this study
V457	P_CUP1_-*MCA1_mut2_-sGFP* (N-terminal extension of MQRRPTRFTI)	pRS316	this study
V458	P_CUP1_-*FFL_19aa_-sGFP* (N-terminal extension of MSLEVYLNYHQRRPTRFTI)	pRS316	this study
V481	P_CUP1_-*FFL-sGFP*	pRS316	this study

Cloning strategies and primer sequences are given in supplemental Table S1.

### Aggregation analysis of Mca1, Sup35 and Rnq1

Cultures for aggregation analysis were grown in appropriate media to mid-log phase and cell lysis was performed as described previously [5], [27]. Protein aggregates were separated by low- and high-speed centrifugation (18,000 and 100,000 g, respectively) and subsequently analyzed by SDS-PAGE and Western blotting using standard techniques. Sup35, Rnq1 and Mca1 proteins were detected using polyclonal anti-Sup35 antibodies [7], polyclonal anti-Rnq1 antibodies [5] and polyclonal anti-Mca1_454_ antibodies (this study), respectively. For preparation of crude cell extracts for Western Blot analysis NaOH lysis was performed.

### Fluorescence microscopy

Cells containing the respective GFP vectors were grown overnight in appropriate selective media. Subsequently, the cultures were diluted into fresh selective media, expression was induced by addition of 150 µM CuSO_4_ and the cells were grown for additional 24 hours. Fluorescence was observed using a Carl Zeiss fluorescence microscope at 100× magnification using a standard FITC filter set.

### Assays for Ade^+^ formation and curing

Mca1-Sup35C fusions were assayed for their Ade^+^ phenotype through the inability of aggregated Sup35 to terminate translation. Accordingly, readthrough of the *ade1–14* (UGA) allele enables adenine biosynthesis and additionally prevents the accumulation of a red pigment.

Mca1-Sup35C fusion proteins were constitutively expressed in a strain lacking the chromosomal *SUP35* gene and growth on media lacking adenine was analyzed after four and 18 days of incubation, respectively. The strains and plasmids used in this study are listed in Table 1 and Table 2.

**Table 2:** List of yeast strains used and constructed in this study.

Strain number	Relevant genotype	Genotype	Reference
Y67	wt	*his3Δ1, leu2Δ0, met15Δ0, ura3Δ0*	BY4741–(EUROSCARF)
Y75	weak [*PSI^+^*][*PIN^+^*]	*MATa, ade1–14, his3-Δ200, leu2.3,112, trp1-289, ura3-52,* weak [*PSI^+^*][*PIN^+^*]	OT55 [31]
Y81	[*psi^−^*][*pin^−^*]	*MATa, ade1–14, his3-Δ200 or 11,15, leu2.3,112, trp1-Δ, ura3-52, lys2*, [*psi^-^*][*pin^−^*] dx. of GT81-1C (Y82) (GuHCl-cured)	GT409 [32]
Y103	*mca1Δ*	*his3Δ1, leu2Δ0, met15Δ0, ura3Δ1, YOR197w::kanMX4*	BY4741–(EUROSCARF)
Y119		*ade1–14/ade1–14, his3-Δ200 (or 11,15)/his3-Δ200, (or 11,15), leu2.3,112/leu2.3,112, trp1-Δ/trp1-Δ, ura3-52,ura3-52, lys2/lys2*	GT81 [25]
Y133	*SUP35+/sup35Δ* [*PSI^+^*]	*ade1-14/ade1–14, his3-Δ200 (or 11,15)/his3-Δ200 (or 11,15), leu2.3,112/leu2.3,112, trp1-Δ/trp1-Δ, ura3-52,ura3-52, lys2/lys2 SUP35+/sup35Δkanmx6* [*PSI^+^*]	this study
Y312	*Δsup35*/V236	*MATa, ade1–14, his3-Δ200 (or 11,15) leu2.3,112, trp1-Δ, ura3-52, lys2, sup35::kanmx; Δsup35*/V236 (p2HG-*SUP35C*)	this study
Y316	*Δsup35*/V124	*MATα, ade1–14, his3-Δ200 (or 11,15), leu2.3,112, trp1-Δ, ura3-52, lys2, sup35::kanmx; Δsup35*/V124 (p2HG-*MCA1N*-*SUP35C*)	this study
Y320	*Δsup35*/V123	*MATα, ade1–14, his3-Δ200(or 11,15), leu2.3,112, trp1-Δ, ura3-52, lys2, sup35::kanmx; Δsup35*/V123 (p2HG-*MCA1-SUP35C*)	this study
Y322	*Δsup35*/V257	*MATa, ade1–14, his3-Δ200(or 11,15), leu2.3,112, trp1-Δ, ura3-52 lys2 sup35::kanmx, Δsup35*/V257 (p2HG-*MCA1C-SUP35C*)	this study

Details for construction of Y133 and primer sequences are given in Materials and Methods.

## Supporting Information

Table S1 (0.06 MB DOC)
