# Supplementary material for: Extra N-Terminal Residues Have a Profound Effect on the Aggregation Properties of the Potential Yeast Prion Protein Mca1
Source: PLoS One. 2010 Mar 29;5(3):e9929. doi: 10.1371/journal.pone.0009929 (PMC2847904; doi:10.1371/journal.pone.0009929)
Supplement: Table S1 — (0.06 MB DOC) [file pone.0009929.s001.doc]

**Supplemental Information**

**Supplemental Table 1.** Cloning strategies and primer sequences of plasmids constructed in this study.

| **Plasmid number** | **Primer sequences (5’ – 3’)** | **Cloning strategy** |
| --- | --- | --- |
| V84 | ggccggatccATGGGGAAGATGAGCCTCGAAG  ccggccgcggTTGGTCTGTACCTTG | PCR product *Bam*HI / *Sac*II in V26 |
| V85 | ggccggatccATGGGGAAGATGAGCCTCGAAG  ccggccgcggCATAATAAATTGCAGATTTACG | PCR product *Bam*HI / *Sac*II in V26 |
| V106 | ccggggatccatgTCTCAATGTACTGGGCG  ccggccgcggCATAATAAATTGCAGATTTACG | PCR product *Bam*HI / *Sac*II in V26 |
| V123 | ggccggatccATGGGGAAGATGAGCCTCGAAG  ccggccgcggCATAATAAATTGCAGATTTACG  ccggccgcggATGTTTGGTGGTAAAGATCACG  ccgggagctcTTACTCGGCAATTTTAACAATTTTACC | PCR products were first cloned into an intermediate vector (pRS316GAL-MCA-SUP35C). This insert was used to clone into V119. |
| V124 | ggccggatccATGGGGAAGATGAGCCTCGAAG  ccggccgcggTTGGTCTGTACCTTG  ccggccgcggATGTTTGGTGGTAAAGATCACG  ccgggagctcTTACTCGGCAATTTTAACAATTTTACC | See V123 for cloning strategy. |
| V236 | ccggggatccATGTTTGGTGGTAAAGATCACG  ccgggagctcTTACTCGGCAATTTTAACAATTTTACC | PCR product *Bam*HI / *Sac*II in V119 |
| V257 | ccggggatccatgTCTCAATGTACTGGGCG  ccggccgcggCATAATAAATTGCAGATTTACG  ccggccgcggATGTTTGGTGGTAAAGATCACG  ccgggagctcTTACTCGGCAATTTTAACAATTTTACC | PCR products in V119 |
| V334 | cgggatccATGTATCCAGGTAGTGGACG  ccggccgcggTTGGTCTGTACCTTG | PCR product *Bam*HI / *Sac*II in V84 |
| V413 | cgggatccATGTATCCAGGTAGTGGACG  CGCGCGAGCTCctgtcccaatcaacagtgatg | PCR product *Bam*HI / *Sac*I in V294 |
| V414 | ggccggatccATGGGGAAGATGAGCCTCGAAG  CGCGCGAGCTCctgtcccaatcaacagtgatg | PCR product *Bam*HI / *Sac*I in V294 |
| V415 | ccggggatccGCCTTGCCTTCTCCAGAGGGTCC  CGCGCGAGCTCctgtcccaatcaacagtgatg | PCR product *Bam*HI / *Sac*I in V294 |
| V454 | cgggatccATGTATCCAGGTAGTGGACG  ccggccgcggCATAATAAATTGCAGATTTACG | PCR product *Bam*HI / *Sac*II in V26 |
| V455 | cgcggatccATGAGCCTCGAAGTTTATCTAAACTACC  ccggccgcggCATAATAAATTGCAGATTTACG | PCR product *Bam*HI / *Sac*II in V26 |
| V456 | cgcggatccATGAGCCTCGAAGTTTATCTAAACTACCACatgtatccaggtagtggacg  ccggccgcggCATAATAAATTGCAGATTTACG | PCR product *Bam*HI / *Sac*II in V26 |
| V457 | cgcggatccatgCAAAGAAGACCGACTAGATTTACAATC  ccggccgcggCATAATAAATTGCAGATTTACG | PCR product *Bam*HI / *Sac*II in V26 |
| V458 | cgggatccATGAGCCTCGAAGTTTATCTAAACTACCACCAAAGAAGACCGACTAGATTTACAATCatggaagacgccaaaaacataaag  TCCCCGCGGtgctcctgctccCACTGCGATCTTTCCGCCC | PCR product *Bam*HI / *Sac*II in V26 |
| V481 | cgggatccATGGAAGACGCCAAAAACATAAAG  TCCCCGCGGtgctcctgctccCACTGCGATCTTTCCGCCC | PCR product *Bam*HI / *Sac*II in V26 |
